# Simultaneous Preparation of Chitin and Flavor Protein Hydrolysates from the By-Products of Shrimp Processing by One-Step Fermentation with *Lactobacillus fermuntum*

**DOI:** 10.3390/molecules28093761

**Published:** 2023-04-28

**Authors:** Jiawei Li, Ru Song, Xiaoyu Zou, Rongbian Wei, Jiaxing Wang

**Affiliations:** 1 Key Laboratory of Health Risk Factors for Seafood of Zhejiang Province, School of Food Science and Pharmacy, Zhejiang Ocean University, Zhoushan 316022, China; ljw19970928@163.com (J.L.); zouxy_job@163.com (X.Z.); 2 School of Chemistry and Bioengineering, Guangxi Normal University for Nationalities, Chongzuo 532200, China; apwapw@126.com; 3 Research Office of Marine Biological Resources Utilization and Development, Zhejiang Marine Development Research Institute, Zhoushan 316021, China

**Keywords:** shrimp by-products, *L. fermuntum*, one-step fermentation, chitin, flavor volatiles, gas chromatography–ion transfer spectrometry

## Abstract

One-step fermentation, inoculated with *Lactobacillus fermentum* (*L. fermentum*) in shrimp by-products, was carried out to obtain chitin and flavor protein hydrolysates at the same time. The fermentation conditions were optimized using response surface methodology, resulting in chitin with a demineralization rate of 89.48%, a deproteinization rate of 85.11%, and a chitin yield of 16.3%. The surface of chitin after fermentation was shown to be not dense, and there were a lot of pores. According to Fourier transform infrared spectroscopy and X-ray diffraction patterns, the fermented chitin belonged to α-chitin. More than 60 volatiles were identified from the fermentation broth after chitin extraction using gas chromatography–ion transfer spectrometry analysis. *L. fermentum* fermentation decreased the intensities of volatile compounds related to unsaturated fatty acid oxidation or amino acid deamination. By contrast, much more pleasant flavors related to fruity and roasted aroma were all enhanced in the fermentation broth. Our results suggest an efficient one-step fermentation technique to recover chitin and to increase aroma and flavor constituents from shrimp by-products.

## 1. Introduction

Chitin (poly-β-[1,4]-N-acetyl-d-glucosamine) is considered to be the second dominant biopolymer in nature after cellulose. Due to its special physicochemical properties, such as good biocompatibility and biodegradability [1,2], excellent mechanical strength and chemical resistance [3], as well as multi-powerful functions, such as antibacterial activity [4], anticancer activity, and antioxidant activity [5], the application of chitin in food technology, sustainable agriculture, the chemical industry, the medical and pharmaceutical industry, etc. has attracted great attention in recent years [3,6,7,8].

During the processing of crustacean aquatic products, a lot of by-products and wastes are generated. Besides nutritional components, such as protein, unsaturated fatty acids, and astaxanthin, these by-products also contain 15–40% of chitin [9]. Therefore, the by-products of crustacean processing become the main source of chitin in industry. The chitin extraction process involves two steps: demineralization (DM) and deproteinization (DP). In conventional extraction methods, concentrated acids and alkali were commonly used to remove calcium carbonate and protein, respectively. However, such chemical extraction technology not only causes serious environmental pollution, but it also changes the natural structure of chitin [10]. Compared to the conventional chemical method, the biotechnological extraction of chitin is well known for being environmentally friendly because organic acids (lactic acid, acetic acid, citric acid, among others) produced by bacterial fermentation can perform the DM stage, and bacterial protease can catalyze the DP process [11]. A green chemistry production process can be assured.

So far, many documents have reported the use of appropriate microbial fermentation to extract chitin from crustaceans. These biological processes can be summarized into three categories: (1) one step fermentation, using single-bacterial fermentation for DM and DP, such as a protease-producing bacterium *Pseudomonas aeruginosa*, which is used for shrimp shell waste fermentation [1]; (2) successive two-step fermentation, using two different bacteria in the DM and DP stages—one bacterium related to protease production and the other one associated with acid production, such as *Serratia marcescens* and *Lactobacillus plantarum*, which are successively used for DP and DM to obtain chitin from shrimp shell powders [12]; and (3) successive co-fermentation with two different bacteria at same time to carry out DM and DP, such as *Bacillus subtilis* (*B. subtilis*) and *Lactobacillus plantarum* (*L. plantarum*) used in bio-extraction of chitin from shrimp shells [13].

In successive two-step fermentation, it is required to remove the protease-producing bacteria before incubation of the acid-producing bacterium. This meant much more time needed for re-sterilization and fermentation. Compared to successive two-step fermentation, the successive co-fermentation showed advantages in DM and DP stages, simultaneously occurring by inoculation of two kinds of bacteria. However, there is no need for repeated sterilization, so this saves time and reduces production costs [13]. However, the selection of coexisting bacteria capable of protease and acid production is time consuming. In many cases, the coexistence of protease- and acid-producing strains is not satisfactory. For example, in a successive co-fermentation system with *B*. *subtilis* and *Acetobacter pasteurianus* to extract chitin, the growth of *Acetobacter pasteurianus*, using ethanol as the carbon source, showed a suppression effect on the growth of *B*. *subtilis* [14]. Therefore, it is necessary to determine when the second bacterium is inoculated for co-fermentation, as well as to optimize the composition of the co-fermentation medium to promote DM efficiency. Among the three categories for chitin extraction by fermentation, one-step fermentation is the simplest method. The efficiency of DM and DP in one-step fermentation can be obtained by the optimization of fermentation parameters, such as initial fermentation pH, fermentation temperature, fermentation time, carbon source addition, etc. [15]. Castro, Guerrero-Legarreta, and Bórquez (2018) reported the DM and DP of chitin from crab biomass reached to 99.6% and 95.3%, respectively, under optimal fermentation conditions by *L. plantarum* fermentation for 60 h [16]. Arbia et al. (2013) optimized the medium composition for enhanced chitin extraction from *Parapenaeus longirostris* by incubation of *Lactobacillus helveticus* for 15 days and obtained 98% of DM and 78% of DP [15]. It was noted that lactic acid bacteria (LAB) demonstrated good prospects for chitin extraction in one-step fermentation. Furthermore, LAB are considered as safe start cultures to promote nutritional and flavor properties in fermented foods [17].

The aim of the present study is to extract chitin from shrimp by-products using one-step fermentation by inoculation of *Lactobacillus fermentum* (*L. fermuntum*), one kind of LAB commonly used in Chinese traditional fermented food processing. Based on the analysis of fermentation parameters, including fermentation time, inoculation amount, initial fermentation pH, shaking rotation speed, fermentation temperature, and glucose content as a carbon source related to DM and DP efficiencies, the fermentation conditions were optimized by means of response surface methodology (RSM). The chitin obtained by *L. fermuntum* fermentation was characterized by field emission scanning electron microscopy (FESEM), Fourier transform infrared spectroscopy (FTIR), and X-ray diffraction (XRD) measurements. In addition, the volatile flavor characteristics of fermentation broth recovered after chitin extraction were evaluated by gas chromatography–ion transfer spectrometry (GC–IMS) analysis. Our findings will provide an efficient and environmentally friendly method for chitin extraction and flavor protein hydrolysates production from shrimp by-products by the one-step fermentation technique.

## 2. Results and Discussion

### 2.1. Effects of Fermentation Conditions on DM and DP Efficiencies

**Figure 1:**
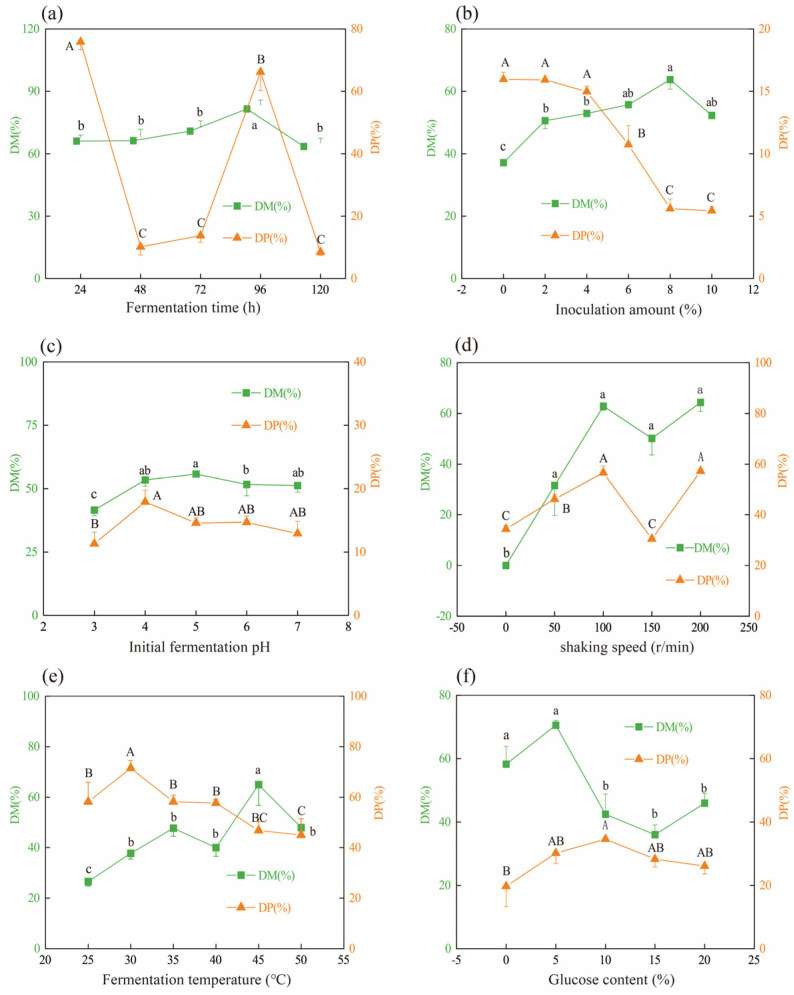
Effects of fermentation conditions on DM and DP of fermented products inoculated with *L. fermuntum.* (**a**) Fermentation time, (**b**) inoculation amount, (**c**) initial fermentation pH, (**d**) shaking speed, (**e**) fermentation temperature, and (**f**) addition of glucose content. Different lower and capital letters indicated significant differences for % DM and % DP among groups (*p* < 0.05), respectively.

It is crucial to allow sufficient contact between the starter culture and the substrate in fermentation to generate the required products [18,19]. After 24 h of fermentation, the DM and DP efficiencies were 66.08% and 75.84%, respectively (Figure 1a). An increase in % DM can be shown as the fermentation time increased and reached a maximum at 96 h. By comparison, dramatic decrease in % DP was observed when the fermentation time ranged from 48 h to 72 h. In this study, the efficiency of DP was determined by measuring the protein content in fermentation broth (see Equation (3)). Therefore, lower % DP meant less protein amount in the fermentation broth. Decreases in % DP in fermentation products when incubated from 48 h to 72 h might be ascribed to proteins further degradation by both endogenous and microbial proteases and peptidases to generate smaller peptides or amino acids. These peptides and amino acids could provide the nutrients needed for the growth of *L. fermentum*. As a result, organic acid production by *L. fermentum* could increase the efficiency of DM. At the same time, the proteolytic enzymes secreted could release proteins from the shrimp shells into the fermentation broth. As shown in Figure 1a, a highest % DP observed at 96 h may be ascribed to the mineral-binding proteins released into fermentation broth, while the decrease in % DP after 96 h of incubation time was likely due to further protein digestion in the fermentation broth.

After 96 h fermentation, the effect of inoculum amount on DM and DP was determined in Figure 1b. In the control group, without inoculum of LAB, the observed % DM and % DP should be ascribed to the fermentation of native microbiota in shrimp wastes. Compared to the non-inoculum, inoculation of *L. fermuntum* increased DM efficiency, and a higher % DM was detected at 8% inoculum, while the latter 10% inoculum did not further improve % DM. This meant the DM efficiency was constant when the inoculation amount of *L. fermuntum* increased from 8% to 10%. Similar to our results, Castro et al. (2018) reported that 10% inoculum of *L. plantarum* on crab shell increased bacteria growth and acid production and promoted the extraction of chitin using lactic fermentation [16]. In this study, decreases in % DP were found to be associated with inoculum increase, since the rapid growth of *L. fermuntum*, initiated by a large inoculation amount, could facilitate the secretion of more proteases to further hydrolyze protein in the fermentation broth into smaller peptides or amino acids, leading to a decrease in % DP.

During LAB fermentation, the proteolytic enzymes produced by inoculated strains, or presenting in the fermentation raw materials themselves, were activated by the low pH of initial fermentation, resulting in hydrolyzed proteins in materials. This was followed by further digestion into smaller peptides or amino acids. The acid environment formed by LAB fermentation could contribute to DM efficiency (mainly calcium carbonate) in shrimp wastes [18]. The initial fermentation pH will affect the growth rate of inoculated LAB and, consequently, its ability to produce organic acids and bacterial protease [20]. In the case of chitin extraction by lactic acid fermentation, using a single bacterial culture, the DM and DP processes occurred simultaneously. The organic acids can proceed to the DM stage, and the bacterial proteases digest protein to obtain chitin [21]. According to the literature, in this case, that is, if LAB are used, the DM and DP have a negative relationship, and the DP rate is usually low because the change in protease configuration under acidic pH environment could affect its activity. Conversely, if proteolytic bacteria are used, the DM rate is low [22,23]. However, as was shown in Figure 1c, the % DM and % DP both increased with elevated initial fermentation pH, increasing from 3.0 to 4.0, whereas they remained constant when the fermentation was started at pH 4.0 through 7.0. Our findings indicated high DM and DP efficiencies on shrimp by-products could be gained by inoculation of single strain *L. fermuntum*. During the process of *L. fermuntum* fermentation started at different initial pHs, the checked pH values of fermentation media altered between pH 6.5 to pH 7.0 (Figure S1). This meant slight inhibitory effect of acidic pH on the activity of proteases in fermentation, which might explain high % DP in this study.

Under static state (0 r/min of shaking speed), the % DM was very low because the raw materials settled at the bottom of the conical flask and did not fully contact with acids produced by *L. fermuntum*, resulting in calcium carbonate not being able to fully dissolve into fermentation. With rotation speed increase, the % DM and % DP reached high levels at 100 r/min (Figure 1d). However, when the rotation speed increased to 150 r/min, the % DP declined to 30.49%. This result could be explained by protein being further digested in fermentation broth by proteases at appropriate rotational speeds. However, too high of a rotation speed could disrupt the interaction between proteases and proteins, thus contributing to reduced protein degradation in the fermentation broth. This might account for the increase in % DP (54.10%) at 200 r/min.

Fermentation temperature will affect the growth rate of LAB and its lactic acid production [16]. When the fermentation temperature ranged from 25 °C to 30 °C, both the % DM and % DP increased accordingly. After that, the % DM remained constant at fermentation temperature from 35 °C to 40 °C (Figure 1e), probably due to the high growth rate of *L. fermuntum*, which is consistent with acid production. The sharp increase in % DM at 45 °C might be related to the easier dissolution of minerals from shrimp shells at an appropriate temperature. However, further increase in fermentation temperature (>45 °C) did not contribute to elevation of the % DM. By contrast, decreases in % DP were detected at fermentation temperatures from 35 °C to 50 °C, especially at 45 °C and 50 °C. Long-term exposure to high temperature may lead to protease inactivation [24], which might be one of the reasons for % DP decreases at 45 °C and 50 °C.

Glucose can act as a carbon source for the growth of LAB, and it can finally be converted into lactic acid [21]. During lactic acid fermentation for chitin extraction, calcium carbonate was dissolved from shrimp shells under acidic conditions, and then it was dissolved in lactic acid to form calcium lactate [16]. Therefore, when glucose is not added, LAB lacks a convertible carbon source, which limits the production of organic acids, such as lactic acid, thus reducing the DM efficiency. As expected, increases in % DM and % DP were detected when the glucose content was added at 5%, compared to no glucose controls (Figure 1f). However, the % DM decreased significantly when the glucose content was more than 5% (*p* < 0.05); meanwhile, the % DP was relatively stable under glucose content, varying from 5% to 20%.

### 2.2. Optimization of Fermentation

In order to obtain suitable fermentation conditions for chitin extraction by inoculation of *L. fermuntum*, based on the results of Figure 1, four variables, including fermentation time, inoculation amount, fermentation temperature, and glucose content with three levels (Table 1), were selected to optimize the fermentation parameters using RSM with % DM and % DP as the responses, respectively.

According to multiple regression analysis of the data in Table 2, the equations for % DM and % DP were listed as follows, respectively.

Y = 80.67 − 0.57A + 3.87B − 0.55C + 22.74D + 0.49AB − 4.75AC − 4.92AD + 9.79BC − 2.68BD − 2.44CD − 5.47A^2^ − 10.42B^2^ − 20.39C^2^ − 12.50D^2^ (for % DM)

Y = 87.24 + 2.74A − 5.90B + 3.15C + 1.00D − 0.30AB + 7.48AC + 0.50AD + 3.18BC − 0.76BD + 0.63CD − 5.44A^2^ + 1.85B^2^ − 51.35C^2^ − 4.68D^2^ (for % DP)

The results of variance analysis of % DM and % DP were shown in Table 3. The model F values of 14.24 and 14.29 for % DM and % DP suggested the significance of the model. Furthermore, a *p* value < 0.0001 for % DM and % DP meant less chance of a “model F value”, which is caused by noise. The model term can be used to explain the variation in the response in the RSM experiment once its *p* value is less than 0.05 [25]. In the case of % DM, the model terms of D, BC, B^2^, C^2^, and D^2^ were all significant (*p* < 0.01 or *p* < 0.05), while C^2^ was the only significant term for the % DP (*p* < 0.01).

Compared to pure error, the *p* values of “lack of fit” for % DM (0.0915) and % DP (0.5183) (*p* > 0.05) indicated that the lack of fit was not significant, so the model constructed by the experiment can be used to describe the observed data. The higher coefficient values of the model (*R*^2^ = 0.9344 for % DM and *R*^2^ = 0.9346 for % DP) also implied that the predicted data were close to the actual ones. Therefore, the model was well adapted to experimental optimization.

**Figure 2:**
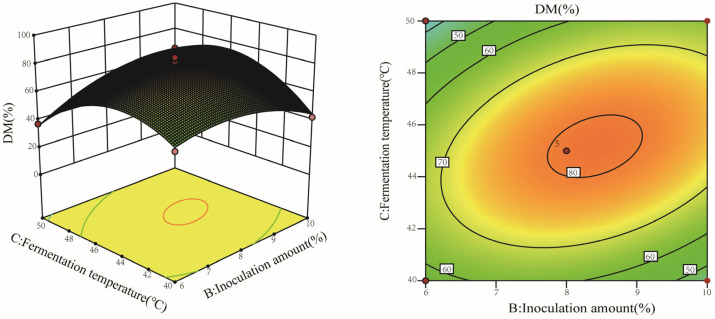
Interaction effect of inoculation amount and fermentation temperature on the DM efficiency.

According to the model, inoculation amount (B) and fermentation temperature (C) produced an interactive effect on the response of % DM (*p* < 0.05). As was shown in Figure 2, at lower levels of inoculation amount, such as 6%, the % DM displayed an increase, coupled with the increase in fermentation temperature, reaching a maximum level at 44 °C. Then, it declined, regardless of the fermentation temperature increase. Similar results were observed at higher levels of inoculation amount (i.e., 10%) when interacting with the fermentation temperature.

Canonical analysis revealed that the optimized fermentation parameters for chitin extraction by incubation with *Lactobacillus* were fermentation time of 95 h, inoculation amount of 7.4%, fermentation temperature of 45 °C, and glucose addition of 8.6%. Three batches of fermentation were performed under the optimal fermentation conditions with the initial fermentation pH of 5.0 and rotation speed of 100 r/min. The actual % DM and % DP were 89.48% and 85.11%, respectively, which were very close to the predicted values of % DM (89.35%) and of % DP (87.35%). Therefore, the mathematical model can be used for the prediction of chitin fermenting extraction. The chitin yield under the optimized fermentation conditions reached 16.3%.

The quality of chitin extracted by *L. fermuntum* fermentation was compared with commercial chitin (CC), which is based on GB1886.312-2020 “National Standards for Food Safety Food Additive chitin” and SC/T3403-2018 “Chitin, Chitosan” (China) in Table 4. The indicators of pH, water content, color, texture, aroma, and impurity of substances were in line with the National Standards for Food Safety Food Additive chitin, and the ash content met the industrial grade chitin standard (≤3.0%).

### 2.3. Characteristics of Fermented Chitin by L. fermuntum Fermentation

#### 2.3.1. Surface Morphology under FESEM Observation

**Figure 3:**
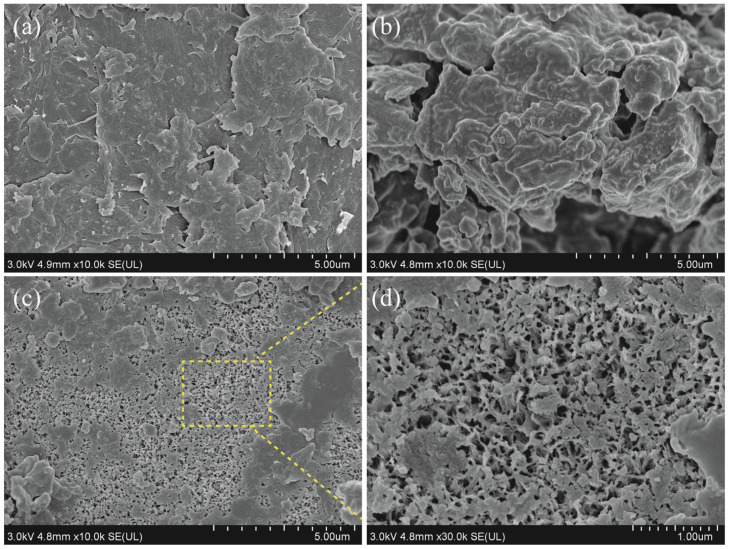
FESEM images of fermented chitin from 95 h of *L. fermuntum* fermentation (SBsL) as compared to chitin samples. (**a**) Commercial chitin (CC); (**b**) shrimp by-products powder (SBs); (**c**,**d**) fermented chitin observed under different magnifications.

Compared to the flakes of CC shown in Figure 3a, rough and clustered surface morphology could be observed on SBs (Figure 3b), and these large aggregates could be associated with the mixture of chitin with protein and inorganic salts. After SBs were fermented by *L. fermuntum* for 95 h, much more noticeable pores appeared on the surface of fermented chitin (Figure 3c). Similar to our studies, Zhang et al. (2021) described many pores that could be apparently observed on the surface of chitin prepared by single fermentation of *B. subtilis* for 3 d [14]. The removal of protein and inorganic salts, such as calcium carbonate and magnesium carbonate from shrimp shells, could be responsible for pore formation on the surface of chitin prepared by the fermentation method. An amplified scanning image of chitin from SBsL was further shown in Figure 3d. The microfiber structure, with dominant pores on the surface, suggested that chitin prepared by *L. fermuntum* fermentation would be very suitable for heavy metal ion adsorption.

#### 2.3.2. FTIR Analysis

**Figure 4:**
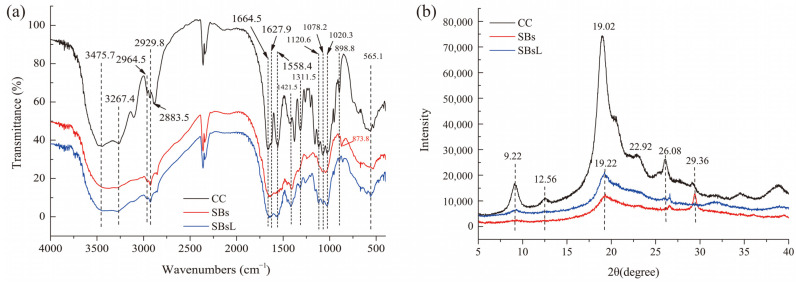
FTIR and XRD spectra of fermented chitin by *L. fermuntum* fermentation as compared to chitin samples. (**a**) FTIR, and (**b**) XRD. Commercial chitin (CC), shrimp by-products (SBs), and fermented chitin were prepared by *L. fermuntum* fermentation for 95 h (SBsL). For clarity, the FTIR spectra of CC and SBsL were shifted up the transmittance (%) by 20% and down the transmittance (%) by 10%, respectively. The XRD pattern of SBs was shifted down in intensity by 3000.

The FTIR spectra of CC, SBs, and SBsL were presented in Figure 4a. CC usually has typical adsorption peaks of a crystalline polysaccharide at 3479 cm^−1^, which are related to OH– stretching vibration. The following adsorption peaks were observed: 3264 cm^−1^ was ascribed to NH– stretching vibration; 2965 cm^−1^, 2927 cm^−1^, and 2883 cm^−1^ were related to CH_3_– or CH_2_– vibration; 1652 cm^−1^ and 1555 cm^−1^ were assigned to amide I and amide II groups; and 1020–1160 cm^−1^ was associated with CO– stretching vibration [14,26,27]. In this study, the absorption peaks, appearing at 3475.7 cm^−1^, 3267.4 cm^−1^, 2964.5 cm^−1^, 2929.8 cm^−1^, 1664.5 cm^−1^, 1558.4 cm^−1^, 1120.6 cm^−1^, 1078.2 cm^−1^, and 1020.3 cm^−1^ in CC and SBsL suggested their typical crystalline polysaccharide structures.

The amide I group in the region of 1630–1700 cm^−1^ was divided into adsorption bands at 1664.5 cm^−1^ and 1627.9 cm^−1^ in CC, which meant that the α-chitin characteristic of CC was used in this study [28,29]. These bands are related to the α-chitin form, and they were also detected in SBsL. In addition, both CC and SBsL had peaks at 1421.5 cm^−1^ (assigned to δ_CH2_), 1311.5 cm^−1^ (assigned to C-N vibration and Amide III of δ_NH_), 898.8 cm^−1^ (assigned to β-configuration in the anomeric center of C1), and 565.1 cm^−1^ (related to γ_C-C_). Obviously, compared to SBs, the FTIR spectrum of SBsL was more similar to that of CC. Although a slight peak at 862.2 cm^−1^ in SB_S_L was close to the typical peak of calcium carbonate at 874 cm^−1^ [28], its intensity was obviously lower than that of SBs at 873.8 cm^−1^. These results implied a tiny amount of calcium carbonate residue in SBsL, which was consistent with the actual DM efficiency of 89.48% in the verification experimental result in RSM.

#### 2.3.3. XRD Analysis

Both CC and the fermented chitin of SBsL showed crystalline reflections at 2θ of 9.22°, 19.02°, 19.22°, and 26.08° (Figure 4b), which were in accordance with previous XRD patterns of α-chitin from shrimp, lobster, crab, and King crab [27]. Calcium carbonate has a specific peak at 2θ around 29.55° [30]. Therefore, the disappearance of 2θ = 29.36° in SBsL confirmed the efficiency of DM by *L. fermuntum* fermentation. In addition, compared to CC, the absence of peaks at 2θ of 12.56° and 22.92° in SBsL might be related to its relatively lower crystallinity because more pore formation reduced the crystallinity. The XRD pattern of SBsL was a supportive consequence to the results of surface structure observation by FESEM (Figure 3c,d).

### 2.4. Volatile Properties of the Fermentation Broth by GC–IMS Analysis

**Figure 5:**
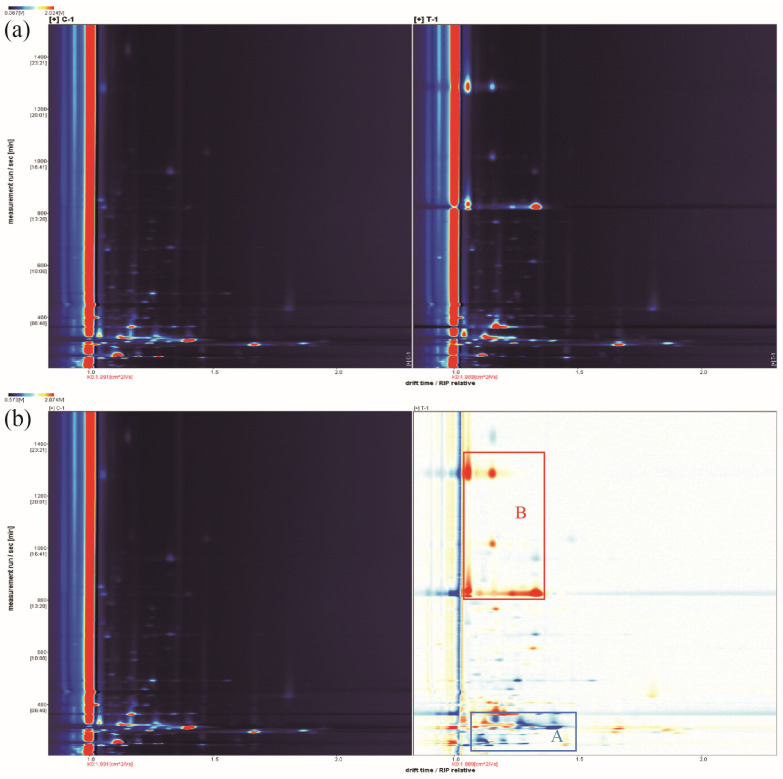
Characteristics of volatile compounds in the fermentation broth of SBs (control group) and SBsL in topographic plots by GC–IMS analysis. (**a**) Original topographic plots; (**b**) difference comparison using SBs as the reference.

The volatile compound change in shrimp by-products before and after *L. fermuntum* fermentation were compared by GC–IMS analysis. In two-dimensional difference profiles (Figure 5a), the flavor properties of SBs and SBsL after chitin extraction were detected with measurement run time, ranging from 200 to 1600 s, as well as drift time/reaction ion peak (RIP), which changed within 1.0 to 2.0.

The red vertical line at abscissa 1.0 in the two-dimensional difference profiles represents the RIP peak (reaction ion peak, after normalization). Each point on both sides of the RIP peak represents a volatile organic compound. The concentration of the substance is marked with white (a lower concentration) or red color (a higher concentration). The darker the color is, the higher the concentration is. These spots (ion peaks), which were detected in Figure 5a with different positions and heights, revealed different volatile profiles of SBs after *L. fermuntum* fermentation.

Taking SBs as the reference, the signal peaks in SBsL were subtracted to obtain the differentiation spectra in detail. The blue area (as shown in area A in Figure 5b) and red area (as shown in area B in Figure 5b) indicated lower and higher concentrations of volatile compounds in SBsL, respectively, compared to SBs. Similarly, the darker the color, the greater the difference between them. Obviously, shrimp by-products that underwent *L. fermuntum* fermentation not only promoted the production of some volatile compounds, but they also reduced the content of some volatile substances. These volatile compounds, detected in the two-dimensional GC–IMS spectra of SBsL, were well separated. Higher intensities of specific volatile compounds (red spots) appeared at the retention time from 200–400 s and 800–1400 s. However, lower intensities of volatile compounds (blue spots) mainly concentrated in the region of 200–400 s retention time.

### 2.5. Qualitative Analysis of Volatile Compounds

**Figure 6:**
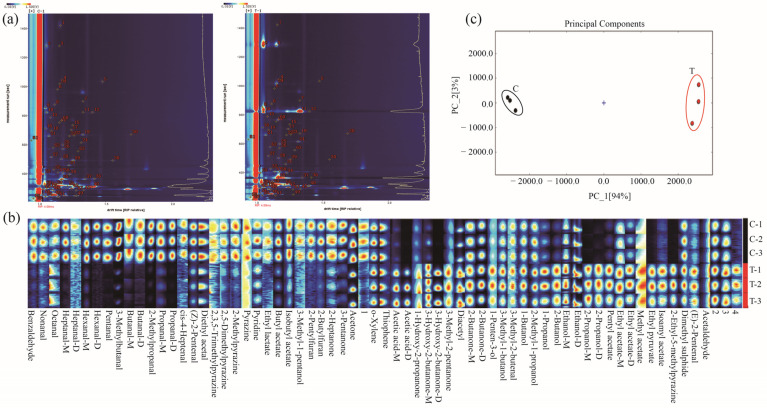
Identification of GC–IMS signals in chromatographic fingerprinting of the fermentation broth of SBs (control group) and SBsL. (**a**) Selected peaks (spots) for identification, each number represents one ion peak (**b**) fingerprinting of volatile compounds, and (**c**) principal component analysis. C and T represented the groups of SBs and SBsL, respectively. Numbers 1, 2, and 3 in (**b**) the image meant detection in triplicate.

A total of 67 ion peaks were detected in SBs and SBsL (Figure 6a), and 63 unique ion signals, including monomer (M) and dimer (D), were identified, as was shown in Table 5. Among the identified volatile compounds, ten volatile compounds, such as acetic acid, 3-hydroxy-2-butanone, heptanal, hexanal, ethanol, 2-butanone, ethyl acetate, butanal, propanal, and 2-propanol, had M and D forms. The changes in each volatile compound were intuitively observed in the fingerprint visualization (Figure 6b). In aquatic products, straight-chain aldehydes, such as acetaldehyde, propionaldehyde, butyraldehyde, hexanal, heptaldehyde, octyl aldehyde, and nonanal, will be produced due to unsaturated fatty acid oxidation or amino acid deamination [31,32]. In this study, high intensities of nonanal, heptanal, hexanal (M and D forms), butanal (M and D forms), and propanal (M and D forms) are present in SBs. Therefore, this could be ascribed to the slight oxidation of lipids because shrimp head is rich in unsaturated fatty acids [33].

During LAB fermentation, organic acids, such as acetic, lactic, citric, succinic, and propionic acids, are generated by the fermentation of carbohydrates [17]. These organic acids can not only prevent spoilage and improve food safety and acceptance, but they can also promote antioxidant effects [34]. As expected, an increase in acetic acid was measured in SBsL. Decreases in straight-chain aldehydes, associated with lipid oxidation in SBsL, should be attributed to acetic acid generated by LAB fermentation. In addition, smaller peptides and free amino acids, as well as short-chain fatty acids or vitamins, generated in fermented products by inoculation of *L. plantarum*, had the capacity to inhibit lipid and protein oxidation to some extent [35,36]. The reduction of aldehyde related to lipid oxidation in SBsL, therefore, could be the result of the combined effects of these functional ingredients. Besides aldehyde compounds, other volatile compounds, such as pyrazines (2,3,5-trimethylpyrazine, 2,5-dimethylpyrazine, 2-methylpyrazine, and pyrazine), associated with a roasted aroma, as well as furans (2-pentylfuran and 2-butylfuran), related to vegetable and fruity aromas [33], had high intensities in SBs. Similarly, the volatile compounds of esters, including isobutyl acetate and butyl acetate, are related to fruity aromas. Acetones of 2-heptanone and acetone are associated with fruity aroma. Additionally, 3-pentanone is related to fermentative flavor. These showed high intensities in the control group of SBs.

All these results indicated that fresh SBs, themselves used in this study for fermentation, had a pleasant flavor. After 95 h of fermentation by *L. fermuntum* incubation, these pleasant volatile compounds in SBs were all significantly decreased or disappeared. However, the ten alcohols, six ketones, and seven esters were all increased in SBsL. Furthermore, the disappearance of the pyridine compound, which is related to a stenchy aroma in SBsL, also suggested that the function of *L. fermuntum* fermentation could be applied to slow down the spoilage of SBs and to promote food flavor.

The aldehydes in SBsL, which were identified with high intensities, such as acetaldehyde and (E)-2-pentenal, were related to the fruity aroma. After *L. fermuntum* fermentation, many more alcohols were detected in SBsL, among which 1-butanol, 2-butanol, and 1-penten-3-ol were correspondingly related to the balsamic, apricot, and meaty aromas. Similarly, more ketone compounds were detected in SBsL because of lipid oxidation or amino acid breakdown via Strecker degradation [33,37,38]. The creamy aroma of 3-hydroxy-2-butanone-D/M might contribute to the pleasant flavor in SBsL. Compared to the SBs control group, many more ester volatile compounds were detected in SBsL, which could be ascribed to the esterification reactions between short-chain fatty acids and alcohols [38]. Among these, five esters were related to the fruity aroma, such as ethyl lactate, pentyl acetate, methyl acetate, ethyl pyruvate, and isoamyl acetate [33,39]. All these esters could play crucial roles regarding the combined flavor of SBsL. Furthermore, high intensity of 2-ethyl-5-methylpyrazine, related to the Maillard reaction [40], could endow a nutty and roasted odor to SBsL products.

These results showed that *L. fermuntum* fermentation changed the original flavor of shrimp by-products and produced a novel, pleasant flavor in fermented products. Based on the GC–IMS data analysis of principle components, the two clusters of SBs and SBsL were distinctly separated (Figure 6c). The high contents of aldehydes, alcohols, ketones, esters, and pyrazines, resulting in the significant aroma differences between SBs and SBsL, may drive the cluster separation. Our findings also indicate that GC–IMS can effectively distinguish the changes in volatile components before and after fermentation in SBs.

## 3. Materials and Methods

### 3.1. Materials

Shrimp (*Penaeus sinensis*) was obtained from Laoqi seafood market in Xincheng district, Zhoushan City, China. The freeze-dried *L. fermuntum* (GIM 1.1796) strain was purchased from Guangdong Microbial Culture Collection Center, Guangzhou City, China. Chitin (CAS1398-61-4, practical grade, molecular mass 203.19) was purchased from Shanghai Aladdin Biochemical Technology Co., Ltd. (Shanghai, China). Bovine serum albumin (BSA) was obtained from Sigma-Aldrich, Inc. (St. Louis, MO, USA). Glucose and other reagents and chemicals utilized were of analytical grade and were purchased from Sinopharm Chemical Reagent Co., Ltd. (Shanghai, China).

### 3.2. Strain Activation and Seed Liquid Preparation

The freeze-dried strain of *L. fermuntum* was activated in MRS medium (5.0 g casein peptone, 2.5 g yeast extract, 2.5 g sodium acetate, 0.5 g Tween 80, 0.1 g magnesium sulfate heptahydrate, 5.0 g beef extract, 10.0 g glucose, 1.0 g citric acid diammonium, 1.0 g potassium dihydrogen phosphate, and 0.0136 g anhydrous manganese sulfate diluted to 500 mL with deionized water) at 37 °C for 24 h. After being transferred twice until the cell density reached 10^7^ CFU/mL, the activated bacterial suspensions (2, 4, 6, 8, and 10 mL) were pipetted into 100 mL of MRS medium, respectively. After 12 h of incubation at 37 °C, the seed liquid (inoculation amounts of 0, 2, 4, 6, 8, and 10%) was used for fermenting.

### 3.3. Fermentation Conditions

Shrimp by-products, including head and shell collected manually, were smashed and used as fermentation substrate. Then, 5.0 g of fermentation substrate, 25 mL of seed liquid, and 10 mL of glucose solution (0, 5, 10, 15, and 20%) were added to a 250 mL conical flask, and the initial fermentation pH was adjusted to pH 3.0, 4.0, 5.0, 6.0, and 7.0. All samples were transferred to a ZQZY-88BV incubator (Shanghai Zhichu Instrument Co., Ltd., Shanghai, China). The fermentation parameters were controlled for fermentation time (24, 48, 72, 96, and 120 h), shaking rotation speed (0, 50, 100, 150, and 200 r/min), and fermentation temperature (25, 30, 35, 40, 45, and 50 °C). To evaluate the effects of the above variables on fermenting, the DM of solid precipitation and DP of fermentation broth were determined.

### 3.4. Demineralization (DM) Efficiency Measurement

The ash content of the solid precipitation collected from the fermentation products was determined according to GB5009.4-2016—“Determination of Ash in Food under National Standards for Food Safety”. In brief, samples were heated in a SX-2-5-12TP muffle furnace (Nanjing Baodu Instrument Co., Ltd. Nanjing, China) at 550 °C ± 25 °C for at least 4 h until reaching constant mass (weight difference ≤ 0.5 mg). The percentage of ash content (W%) was determined according to Equation (1).

(1) $$W\;(\%)=\frac{m_{1}-m_{2}}{m_{3}-m_{2}}\times 100$$

where m_1_, m_2_, and m_3_ represented the weights (g) of crucible and ash, crucible, and crucible and sample, respectively.

The percentage of DM (%) was calculated according to Equation (2).

(2) $$\mathrm{DM}\;(\%)=\frac{W_{0}\times M_{0}-W_{1}\times M_{1}}{W_{0}\times M_{0}}\times 100$$

where W_0_ and W_1_ represented ash content percentages of raw and fermented samples, respectively. M_0_ and M_1_ represented the weights of the raw and solid phases of the fermented samples, respectively.

### 3.5. Deproteinization (DP) Efficiency Measurement

The protein concentration of fermentation broth was measured using Bradford’s (1976) method, with BSA as the standard [41]. In brief, 1 mL of fermentation broth dilution was blended with 5 mL of Coomassie Brilliant Blue (G250). After 5 min of reaction, the absorbance was measured at 630 nm using an UV-spectrophotometer (Shanghai Jinghua Technology Instrument Co., Ltd., Shanghai, China). The absorbance (y) of the BSA solution, with a concentration gradient (0.1–1.0 μg/mL) (x), was determined at the same reacting conditions to obtain the protein calibration curve (y = 0.0015x + 0.0867, *R*^2^ = 0.9835). The protein concentration (μg/mL) of fermentation broth was calculated according to the protein calibration curve, and the percentage of DP was calculated by Equation (3).

(3) $$\mathrm{DP}\;(\%)=\frac{\mathrm{PV}-P_{0}V_{0}}{P_{0}V_{0}}\times 100$$

where P_0_ and P represent the protein concentration (μg/mL) of pre-fermentation and post-fermentation broth, respectively. V_0_ and V represented the pre-fermentation and post-fermentation broth volume (mL), respectively.

### 3.6. Optimization of Fermentation Conditions

Based on the results of single factors, the Box-Behnken design of RSM was used to optimize the fermentation conditions for four factors, including A—fermentation time (h), B—inoculation amount (%), C—fermentation temperature (°C), and D—carbon source of glucose content (%) (for variables and levels, see Table 1). All 29 designed experiments were performed (Box-Behnken design in Table 2), and the resulting data were fitted with the following equation.

Y = β_0_ + β_1_A + β_2_B + β_3_C + β_4_D + β_12_AB + β_13_AC + β_14_AD + β_23_BC + β_24_BD + β_34_CD + β_11_A^2^ + β_22_B^2^ + β_33_C^2^ + β_44_D^2^

where Y was the dependent variable (% DM or % DP); β_0_ was the constant; β_1_, β_2_, β_3_, and β_4_ were the linear regression coefficients; β_12_, β_13_, β_14_, β_23_, β_24_, and β_34_ were the cross-product regression coefficients; β_11_, β_22_, β_33_, and β_44_ were the quadratic regression coefficients, respectively. The mathematical analyses of the results of Table 2 were carried out using Design Expert Software (version 10, trial Statease Inc., Silicon Valley, CA, USA) and predicted optima A, B, C, and D. Finally, three batches of fermentation products were prepared under the optimal fermentation conditions, and the actual % DM and % DP were compared with their predicted responses so as to verify the optimized fermentation conditions. The fermented shrimp by-products, optimized by *L. fermuntum* fermentation, were named SBsL. The generalized procedure for SBsL preparation is shown in Figure S2.

### 3.7. Chitin Quality Evaluation

The quality of chitin derived from SBsL was evaluated according to GB1886.312-2020—“National Standards for Food Safety Food Additive chitin” and SC/T3403-2018—“Chitin, Chitosan”. Sensory characteristics, including color, texture, aroma, and whether presence of foreign matter was detected, were described.

The pH of SBsL solution (10 g/L) was measured at room temperature using a PHB-4pH meter (REX, Shanghai INESA Scientific Instrument Co., Ltd., Shanghai, China). The moisture content of SBsL was determined after being heated at 101–105 °C in a GZX-9030 electric oven (Shanghai Boxun Industrial Co., Ltd., Shanghai, China) until reaching constant mass (weight difference ≤ 2 mg). The percentage of ash content of SBsL was measured according to the method described in 3.4.

### 3.8. Characteristics of Fermented Chitin

Before and after fermentation, the solid residues collected were repeatedly washed with distilled water to neutral, and then they were freeze-dried and sprayed with gold. The surface morphology of chitin by *L. fermuntum* fermentation or commercial chitin and un-fermented powder was observed by a SU8020 FESEM (Hitachi, Tokyo, Japan) under accelerating voltage of 3.0 kV. Freeze-dried samples were thoroughly mixed with KBr at a ratio of 1:100 (*w*/*w*), grinded evenly, and then compressed into pellets. FTIR spectra were recorded by an IRAffinity spectrometer (Shimadzu Beijing Branch, Beijing, China) from 400–4000 cm^−1^ at a 2 cm^−1^ resolution. XRD patterns of samples were measured by a MiniFlex600 X-ray diffractometer (Rigaku Beijing Corporation, Beijing, China) using Cu-Kα radiation in the region of 2θ from 5° to 40° at a scanning rate of 0.02°/s.

### 3.9. Flavor Characteristics of Fermentation Broth

After filtration of SBs or SBsL to remove solids, the fermentation broth left was used for volatile property assessment using a GC–IMS flavor analyzer (FlavourSpec^®^, Dortmund, Germany), according to our previous study [33], with few modifications. In brief, after 20 min of incubation at 50 °C under 500 rpm in head-space vial, 0.5 mL of sample was automatically injected by a heated syringe (85 °C) into GC–IMS. The separation was performed on a WAX capillary column (15 m × 0.53 mm ID, RESTEK, USA) (60 °C), using nitrogen (purity of 99.99%) as the carrier gas, within 30 min, under the following conditions: 2 mL/min for 2 min; then, there was a linear increase to 10 mL/min at 10 min; this progressed to 100 mL/min at 20 min; and, finally 100 mL/min were retained for 10 min. Nitrogen was used as the IMS drift gas at a flow rate of 150 mL/min, and the IMS temperature was set to 45 °C. Each group was performed in triplicate.

In IMS profiles, the neutral gas phase under atmospheric pressure can separate trace chemicals into ionized compounds and facilitate rapid identification of isobaric and isomeric compounds [42]. In this study, the data of GC–IMS were analyzed for volatile compounds, using the instrument supporting analysis software Vocal and three specialized plug-ins, including Reporter, Gallery Plot, and Dynamic Principal Component Analysis (PCA). Based on the National Institute of Standards and Technology (NIST) and IMS databases, Vocal software was used for qualitative and quantitative analysis of detected volatile compound ions. Three- and two-dimensional topographic plots were directly compared using the Reporter plug-in. The topographic plot signals of the SBsL group were obtained after the ones of the SB_S_ group were subtracted. The Gallery plot plug-in was used to generate volatile compounds’ profile fingerprints. The cluster analysis was performed to determine the types of samples by dynamic PCA plug-in.

### 3.10. Statistic Analysis

Each experiment was performed in triplicate, and the experimental results were expressed as mean ± standard deviation (*n* = 3). Statistical analyses were performed using Origin software and SPSS 26. *p* < 0.05 was considered statistically significant.

## 4. Conclusions

In conclusion, the LAB of *L. fermuntum* can be used to ferment shrimp by-products to extract chitin and to produce flavor fermentation at the same time. Under the optimum fermentation conditions, industry grade chitin, with 16.3% yield and α-chitin property, was obtained with DM and DP efficiencies of 89.48% and 85.11%. In addition, novel pleasant flavor compounds appeared in the fermentation products after chitin extraction. Some typical volatile compounds, such as esters, furans, and pyrazines, were related to fruity, roasted, or nutty aromas, thus showing high intensities in the fermentation. This study provides a simple, efficient, and green approach for large-scale production of chitin and flavor fermentation from shrimp by-products by one-step *L. fermuntum* fermentation. The fermented chitin, with porous surface, obtained in this study, indicates its potential application prospects as an adsorbent, such as for adsorbing harmful substances. In addition, the fermentation broth collected after chitin preparation might be used to develop flavoring seasonings. The next work should focus on the further purification or modification of chitin to improve its quality so as to broaden the application prospect of fermented chitin in many fields.

## Figures and Tables

**Table 1:** Independent variables and their coded and actual values in the Box-Behnken design of response surface methodology.

Code	A Fermentation Time (h)	B Inoculation Amount (%)	C Temperature (°C)	D Glucose Content (%)
−1	72	6	40	0
0	96	8	45	5
+1	120	10	50	10

**Table 2:** Experimental design and responses of the independent variables to fermentation parameters.

No	A	B	C	D	DM (%)	DP (%)
1	72	6	45	5	63.59	92.22
2	120	6	45	5	33.41	25.44
3	72	10	45	5	37.38	37.76
4	120	10	45	5	53.79	49.03
5	96	8	40	0	78.27	87.58
6	96	8	50	0	75.04	96.99
7	96	8	40	10	87.61	78.18
8	96	8	50	10	89.27	78.04
9	72	8	45	0	64.98	31.80
10	120	8	45	0	70.88	71.29
11	72	8	45	10	84.00	90.63
12	120	8	45	10	42.24	30.34
13	96	6	40	5	19.13	93.28
14	96	10	40	5	83.87	82.68
15	96	6	50	5	31.30	61.35
16	96	10	50	5	55.49	31.01
17	72	8	40	5	45.45	74.20
18	120	8	40	5	56.06	19.48
19	72	8	50	5	37.17	33.39
20	120	8	50	5	84.55	89.57
21	96	6	45	0	63.82	91.96
22	96	10	45	0	33.60	91.82
23	96	6	45	10	61.19	35.06
24	96	10	45	10	59.66	30.61
25	96	8	45	5	83.75	92.88
26	96	8	45	5	41.22	49.00
27	96	8	45	5	81.50	71.42
28	96	8	45	5	67.19	24.58
29	96	8	45	5	68.69	72.74

**Table 3:** ANOVA for the response surface quadratic model of decalcification rate and deproteinization.

Source	Sum of Squares		*df*	Mean Square		*F* Value		*p* Value		Significance	
	DM	DP	DM or DP	DM	DP	DM	DP	DM	DP	DM	DP
Model	10,400.88	19,011.39	14	742.92	1357.96	14.24	14.29	<0.0001	<0.0001	**	**
A	3.88	89.87	1	3.88	89.87	0.074	0.95	0.7892	0.3473		
B	179.57	417.25	1	179.57	417.25	3.44	4.39	0.0848	0.0548		
C	3.61	118.69	1	3.61	118.69	0.069	1.25	0.7964	0.2825		
D	6202.56	11.88	1	6202.56	11.88	118.85	0.13	<0.0001	0.7289	**	
AB	0.96	0.35	1	0.96	0.35	0.018	3.726 × 10^−3^	0.8940	0.9522		
AC	90.35	223.80	1	90.35	223.80	1.73	2.36	0.2094	0.1472		
AD	96.73	0.99	1	96.73	0.99	1.85	0.010	0.1949	0.9201		
BC	383.18	40.51	1	383.18	40.51	7.34	0.43	0.0169	0.5244	*	
BD	28.78	2.31	1	28.78	2.31	0.55	0.024	0.4700	0.8783		
CD	23.81	1.60	1	23.81	1.60	0.46	0.017	0.5104	0.8986		
A^2^	194.03	192.24	1	194.03	192.24	3.72	2.02	0.0744	0.1768		
B^2^	704.19	22.10	1	704.19	22.10	13.49	0.23	0.0025	0.6370	**	
C^2^	2697.92	17106.38	1	2697.92	17,106.38	51.70	180.02	<0.0001	<0.0001	**	**
D^2^	1013.41	142.01	1	1013.41	142.01	19.42	1.49	0.0006	0.2417	**	
Residual	730.64	1330.36	14	52.19	95.03						
Lack of fit	666.36	967.98	10	66.64	96.80	4.15	1.07	0.0915	0.5183		
Pure error	64.29	362.37	4	16.07	90.59						
Cor total	11,131.52	20,341.75	28		1357.96						

R^2^ = 0.9344 for % DM and R^2^ = 0.9346 for % DP. ** and * represent *p* < 0.01 and *p* < 0.05, respectively.

**Table 4:** Quality evaluation of chitin extracted by *L. fermentum* fermentation.

Index	Standard	Obtained Chitin
pH	6.5–8.5	6.84 ± 0.32
Water (%)	Industry grade ≤ 12.0	8.79 ± 2.39
	Food grade ≤ 10.0	
Ash (%)	Industry grade ≤ 3.0	2.27 ± 0.84
	Food grade ≤ 1.0	
Color	White to light yellow or light red	Light red
Texture	Flaky or powder	Powder
Aroma	Own characteristic smell and no peculiar smell	Own characteristic smell and no peculiar smell
Impurity substance	No foreign impurities visible in normal vision	No foreign impurities visible in normal vision

**Table 5:** Identification of 63 unique ion signals in shrimp processing by-products and fermented products by incubation of *L. fermentum* through GC–IMS analysis.

Category	Count	CAS#	Compound	Rt [sec]	Dt [RIPrel]	Comment	Odor Type
Aldehydes	1	C100527	Benzaldehyde	1421.885	1.16385		nutty
	5	C124196	Nonanal	1032.99	1.48097		balsamic
	12	C124130	Octanal	824.492	1.40603		grassy
	18	C107868	3-Methyl-2-butenal	693.554	1.09854		none
	19	C111717	Heptanal	669.295	1.33343		fatty
	20	C111717	Heptanal	669.295	1.69629		fatty
	27	C1576869	(Z)-2-Pentenal	515.914	1.0953		potato
	29	C66251	Hexanal	491.654	1.26053	Monomer	fruity
	30	C66251	Hexanal	491.654	1.5586	Dimer	fruity
	36	C110623	Pentanal	365.663	1.4209		fruity
	40	C590863	3-Methylbutanal	310.884	1.4047		fatty
	43	C105577	Diethyl acetal	294.936	1.0356		vegetable
	46	C123728	Butanal	284.79	1.11394	Monomer	floral
	47	C123728	Butanal	284.79	1.28245	Dimer	floral
	49	C78842	Isobutanal	249.277	1.28393		floral
	50	C123386	Propanal	243.189	1.05038	Monomer	earthy
	51	C123386	Propanal	243.189	1.14794	Dimer	earthy
	53	C75070	Acetaldehyde	215.794	0.97795		fruity
	62	C1576870	(E)-2-Pentenal	578.331	1.10772		fruity
Alcohols	7	C589355	3-Methyl-1-pentanol	899.421	1.33494		roasted
	14	C6728310	cis-4-Heptenal	752.821	1.15432		Grassy, fatty
	17	C123513	3-Methyl-1-butanol	701.722	1.24553		alcoholic pungent
	25	C71363	1-Butanol	598.865	1.18277		balsamic
	28	C78831	2-Methyl-1-propanol	501.828	1.17305		winey
	32	C71238	1-Propanol	426.702	1.11474		musty
	38	C64175	Ethanol	320.274	1.0467	Monomer	alcoholic
	39	C64175	Ethanol	321.84	1.1277	Dimer	alcoholic
	55	C67630	2-Propanol	313.876	1.08734	Monomer	woody
	56	C67630	2-Propanol	314.552	1.21741	Dimer	woody
	57	C78922	2-Butanol	407.561	1.14646		apricot
	61	C616251	1-Penten-3-ol	629.46	0.94306		Meaty, vegetable
Ketones	9	C116096	1-Hydroxy-2-propanone	854.898	1.05056		pungent sour
	10	C513860	3-Hydroxy-2-butanone	823.406	1.06017	Monomer	creamy
	11	C513860	3-Hydroxy-2-butanone	824.492	1.33494	Dimer	creamy
	23	C110430	2-Heptanone	663.817	1.26539		fruity
	35	C431038	Diacetyl	362.532	1.17305		creamy
	37	C96220	3-Pentanone	364.098	1.34963		fermentative
	41	C78933	2-Butanone	301.362	1.06221	Monomer	camphor
	42	C78933	2-Butanone	302.038	1.24846	Dimer	camphor
	48	C67641	Acetone	253.674	1.11542		fruity
	58	C565617	3-Methyl-2-pentanone	406.208	1.18194		none
Esters	6	C97643	Ethyl lactate	979.78	1.14279		fruity
	24	C628637	Pentyl acetate	614.516	1.32047		fruity
	31	C123864	Butyl acetate	458.787	1.22651		fruity
	34	C110190	Isobutyl acetate	396.965	1.23299		fruity
	44	C141786	Ethyl Acetate	289.186	1.09916	Monomer	grassy
	45	C141786	Ethyl Acetate	288.51	1.33862	Dimer	grassy
	54	C79209	Methyl acetate	258.747	1.0356		fruity
	60	C617356	Ethyl pyruvate	766.545	1.17296		fruity
	63	C123922	Isoamyl acetate	556.101	1.29723		fruity
Amines	33	C110021	Thiophene	399.313	1.0386		garlic
	52	C75183	Dimethyl sulfide	229.999	0.96021		sulfurous
Furans	15	C3777693	2-Pentylfuran	735.446	1.25808		vegetable
	26	C4466244	2-Butylfuran	571.475	1.18277		fruity
Pyrazines	4	C14667551	2,3,5-Trimethylpyrazine	1044.935	1.1793		roasted
	8	C123320	2,5-Dimethylpyrazine	875.531	1.12166		roasted
	13	C109080	2-Methylpyrazine	788.657	1.08899		roasted
	16	C290379	Pyrazine	710.47	1.05056		roasted
	59	C13360640	2-Ethyl-5-methylpyrazine	1017.703	1.15876		nutty, roasted
Benzenes	21	C95476	o-Xylene	659.904	1.07748		none
Pyridines	22	C110861	Pyridine	659.122	1.23299		stenchy
Acids	2	C64197	Acetic acid	1277.917	1.05603		pungent sour
	3	C64197	Acetic acid	1278.427	1.15468		pungent sour

## Data Availability

Data will be made available upon request.

## Supplementary Materials

The following supporting information can be downloaded at: https://www.mdpi.com/article/10.3390/molecules28093761/s1, Figure S1: Effect of initial fermentation pH on the pH changes of fermentation broth during fermenting. Letter “a” represents *p* > 0.05. Figure S2: The generalized procedure for SBsL preparation.

## References

[1] Sedaghat F., Yousefzadi M., Toiserkani H., Najafipour S. (2017). Bioconversion of shrimp waste *Penaeus merguiensis* using lactic acid fermentation: An alternative procedure for chemical extraction of chitin and chitosan. Int. J. Biol. Macromol..

[2] Mao X., Zhang J., Kan F., Gao Y., Lan J., Zhang X., Hu Z., Li Y., Lin H. (2013). Antioxidant production and chitin recovery from shrimp head fermentation with *Streptococcus thermophilus*. Food Sci. Biotechnol..

[3] Lv J., Lv X., Ma M., Oh D.H., Jiang Z., Fu X. (2023). Chitin and chitin-based biomaterials: A review of advances in processing and food applications. Carbohyd. Polym..

[4] Abdel-Mohsen A.M., Jancar J., Massoud D., Fohlerova Z., Elhadidy H., Spotz Z., Hebeish A. (2016). Novel chitin/chitosan-glucan wound dressing: Isolation, characterization, antibacterial activity and wound healing properties. Int. J. Pharm..

[5] Singh A., Dutta P., Kumar H., Kureel A.K., Rai A.K. (2018). Synthesis of chitin-glucan-aldehyde-quercetin conjugate and evaluation of anticancer and antioxidant activities. Carbohyd. Polym..

[6] Hou F., Gong Z., Jia F., Cui W., Song S., Zhang J., Wang Y., Wang W. (2023). Insights into the relationships of modifying methods, structure, functional properties and applications of chitin: A review. Food Chem..

[7] Giraldo J.D., Garrido-Miranda K.A., Schoebitz M. (2023). Chitin and its derivatives: Functional biopolymers for developing bioproducts for sustainable agriculture—A reality?. Carbohyd. Polym..

[8] Sirajudheen P., Poovathumkuzhi N.C., Vigneshwaran S., Chelaveettil B.M., Meenakshi S. (2021). Applications of chitin and chitosan based biomaterials for the adsorptive removal of textile dyes from water—A comprehensive review. Carbohyd. Polym..

[9] Kurita K. (2006). Chitin and chitosan: Functional biopolymers from marine crustaceans. Mar. Biotechnol..

[10] Khanafari A., Marandi R., Sanatei S. (2008). Recovery of chitin and chitosan from shrimp waste by chemical and microbial methods. Iran. J. Environ. Health Sci. Eng..

[11] Rao M.S., Muñoz J., Stevens W.F. (2000). Critical factors in chitin production by fermentation of shrimp biowaste. Appl. Microbiol. Biot..

[12] Zhang H., Jin Y., Deng Y., Wang D., Zhao Y. (2012). Production of chitin from shrimp shell powders using *Serratia marcescens B742* and *Lactobacillus plantarum ATCC 8014* successive two-step fermentation. Carbohyd. Res..

[13] Zhang Q., Xiang Q., Li Y. (2022). One-step bio-extraction of chitin from shrimp shells by successive co-fermentation using *Bacillus subtilis* and *Lactobacillus plantarum*. Innov. Food Sci. Emerg. Technol..

[14] Zhang Q., Wang L., Liu S., Li Y. (2021). Establishment of successive co-fermentation by *Bacillus subtilis* and *Acetobacter pasteurianus* for extracting chitin from shrimp shells. Carbohyd. Polym..

[15] Arbia W., Adour L., Amrane A., Lounici H. (2013). Optimization of medium composition for enhanced chitin extraction from *Parapenaeus longirostris* by *Lactobacillus helveticus* using response surface methodology. Food Hydrocoll..

[16] Castro R., Guerrero-Legarreta I., Bórquez R. (2018). Chitin extraction from Allopetrolisthes punctatus crab using lactic fermentation. Biotechnol. Rep..

[17] de Souza E.L., de Oliveira K.Á., de Oliveira M.E. (2023). Influence of lactic acid bacteria metabolites on physical and chemical food properties. Curr. Opin. Food Sci..

[18] Xu Y., Gallert C., Winter J. (2008). Chitin purification from shrimp wastes by microbial deproteination and decalcification. Appl. Microbiol. Biotechnol..

[19] Gunawan S., Aparamarta H.W., Darmawan R., Zarkasie I.M., Prihandini W.W. (2018). Effect of initial bacterial cells number and fermentation time on increasing nutritive value of sago flour. Malays. J. Fundam. Appl. Sci..

[20] Namasivayam E., Ravindar J., Mariappan K., Akil J., Kumar M., Jayaraj R.L. (2011). Production of extracellular pectinase by *Bacillus cereus* isolated from market solid waste. J. Bioanal. Biomed..

[21] Sixto-Berrocal A.M., Vázquez-Aldana M., Miranda-Castro S.P., Martínez-Trujillo M.A., Cruz-Díaz M.R. (2023). Chitin/chitosan extraction from shrimp shell waste by a completely biotechnological process. Int. J. Biol. Macromol..

[22] Sorokulova I., Krumnow A., Globa L., Vodyanoy V. (2009). Efficient decomposition of shrimp shell waste using *Bacillus cereus* and *Exiguobacterium acetylicum*. J. Ind. Microbiol. Biotechnol..

[23] Cahyaningtyas H.A.A., Suyotha W., Cheirsilp B., Prihanto A.A., Yano S., Wakayama M. (2022). Optimization of protease production by *Bacillus cereus HMRSC30* for simultaneous extraction of chitin from shrimp shell with value-added recovered products. Environ. Sci. Pollut. Res..

[24] Zhang K., Wei R., Song R. (2019). Extraction of cathepsin D-like protease from neon flying squid (*Ommastrephes bartramii*) viscera and application in antioxidant hydrolysate production. Biomolecules.

[25] Song R., Wei R., Zhang B., Wang D. (2012). Optimization of the antibacterial activity of Half-fin anchovy (*Setipinna taty*) hydrolysates. Food Bioprocess. Technol..

[26] Dong Q., Qiu W., Li L., Tao N., Wang A.L., Deng S., Jin Y. (2023). Extraction of chitin from white shrimp (*Penaeus vannamei*) shells using binary ionic liquid mixtures. J. Ind. Eng. Chem..

[27] Cárdenas G., Cabrera G., Taboada E., Miranda S.P. (2004). Chitin characterization by SEM, FTIR, XRD, and ^13^C cross polarization/mass angle spinning NMR. J. Appl. Polym. Sci..

[28] Li S., Tang S., Mo R., Li J., Chen L. (2023). Effects of NaCl curing and subsequent fermentation with *Lactobacillus sakei* or *Lactobacillus plantarum* on protein hydrolysis and oxidation in yak jerky. LWT-Food Sci. Technol..

[29] Jang M.K., Kong B.G., Jeong Y.I., Lee C.H., Nah J.W. (2004). Physicochemical characterization of α-chitin, β-chitin, and γ-chitin separated from natural resources. J. Polym. Sci. A Polym. Chem..

[30] Li Z., Li M.C., Liu C., Liu X., Lu Y., Zhou G., Liu C., Mei C. (2023). Microwave-assisted deep eutectic solvent extraction of chitin from crayfish shell wastes for 3D printable inks. Ind. Crop. Prod..

[31] Xu Y., Liu Y., Jiang C., Zhang C., Li X., Zhu D., Li J. (2014). Determination of volatile compounds in turbot (*Psetta maxima*) during refrigerated storage by headspace solid-phase microextraction and gas chromatography-mass spectrometry. J. Sci. Food Agric..

[32] Zeng X., Xia W., Jiang Q., Xu Y., Fan J. (2017). Contribution of mixed starter cultures to flavor profile of Suanyu-A traditional Chinese low-salt fermented whole fish. J. Food Process. Preserv..

[33] Xu Y., Song R., Jia Z., Wei R., Wang J., Sun J. (2023). Effect of *Bacillus subtilis* (*Bacillus subtilis* subsp.) inoculation on the fermentation characteristics of Penaeus sinensis by-products: Protease activity and volatile property. LWT-Food Sci. Technol..

[34] Bangar S.P., Suri S., Trif M., Ozogul F. (2022). Organic acids production from lactic acid bacteria: A preservation approach. Food Biosci..

[35] Xu H., Xiao N., Xu J., Guo Q., Shi W. (2022). Effect of *Lactobacillus plantarum* and flavourzyme on physicochemical and safety properties of grass carp during fermentation. Food Chem. X.

[36] Yang F., Xia W.S., Zhang X.W., Xu Y.S., Jiang Q.X. (2016). A comparison of endogenous and microbial proteolytic activities during fast fermentation of silver carp inoculated with *Lactobacillus plantarum*. Food Chem..

[37] Sun S.M., Chung G.H., Shi T.S. (2012). Volatile compounds of the green alga *Capsosiphon fulvescens*. J. Appl. Phycol..

[38] Sha K., Lang Y.M., Sun B.Z., Su H.W., Li H.P., Zhang L., Lei Y.H., Li H.B., Zhang Y. (2017). Changes in lipid oxidation, fatty acid profile and volatile compounds of traditional kazakh dry-cured beef during processing and storage. J. Food Process. Preserv..

[39] Kahle K., Preston C., Richling E., Heckel F., Schreier P. (2005). On-line gas chromatography combustion/pyrolysis isotope ratio mass spectrometry (HRGC-C/P-IRMS) of major volatiles from pear fruit (*Pyrus communis*) and pear products. Food Chem..

[40] Yang P., Wang H., Cao Q., Song H., Xu Y., Lin Y. (2023). Aroma-active compounds related to Maillard reaction during roasting in Wuyi Rock tea. J. Food Compos. Anal..

[41] Bradford M.M. (1976). A rapid and sensitive method for the quantitation of microgram quantities of protein utilizing the principle of protein binding. Anal. Biochem..

[42] Hernández-Mesa M., Ropartz D., García-Campaña A.M., Rogniaux H., Dervilly-Pinel G., Le Bizec B. (2019). Ion mobility spectrometry in food analysis: Principles, current applications and future trends. Molecules.

