# Supplementary figures and images for: Simultaneous Preparation of Chitin and Flavor Protein Hydrolysates from the By-Products of Shrimp Processing by One-Step Fermentation with Lactobacillus fermuntum
Source: Molecules. 2023 Apr 28;28(9):3761. doi: 10.3390/molecules28093761 (PMC10179846; doi:10.3390/molecules28093761)

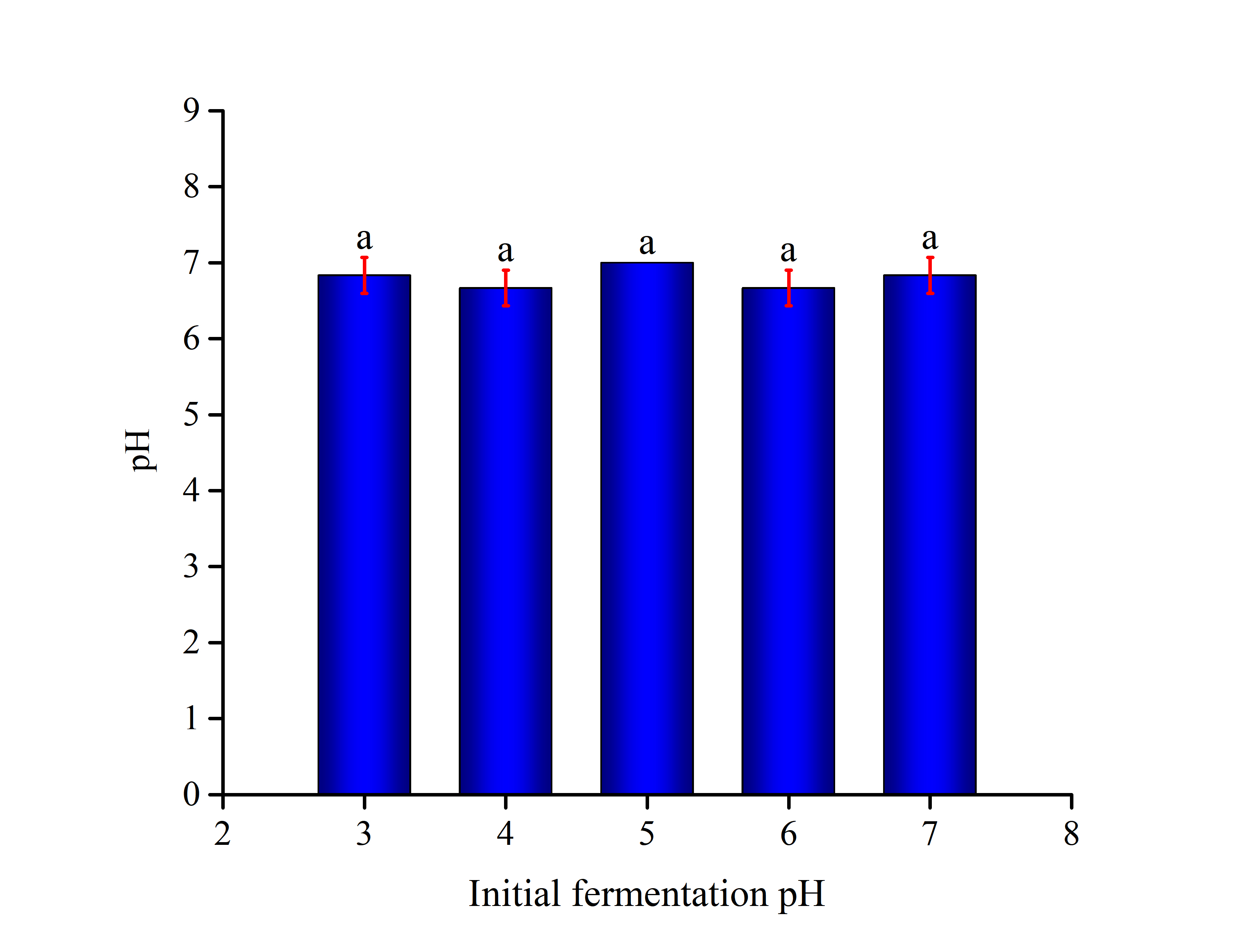

Supplement: Supplementary file 1 [file molecules-28-03761-s001.zip › Figure S1.jpg]

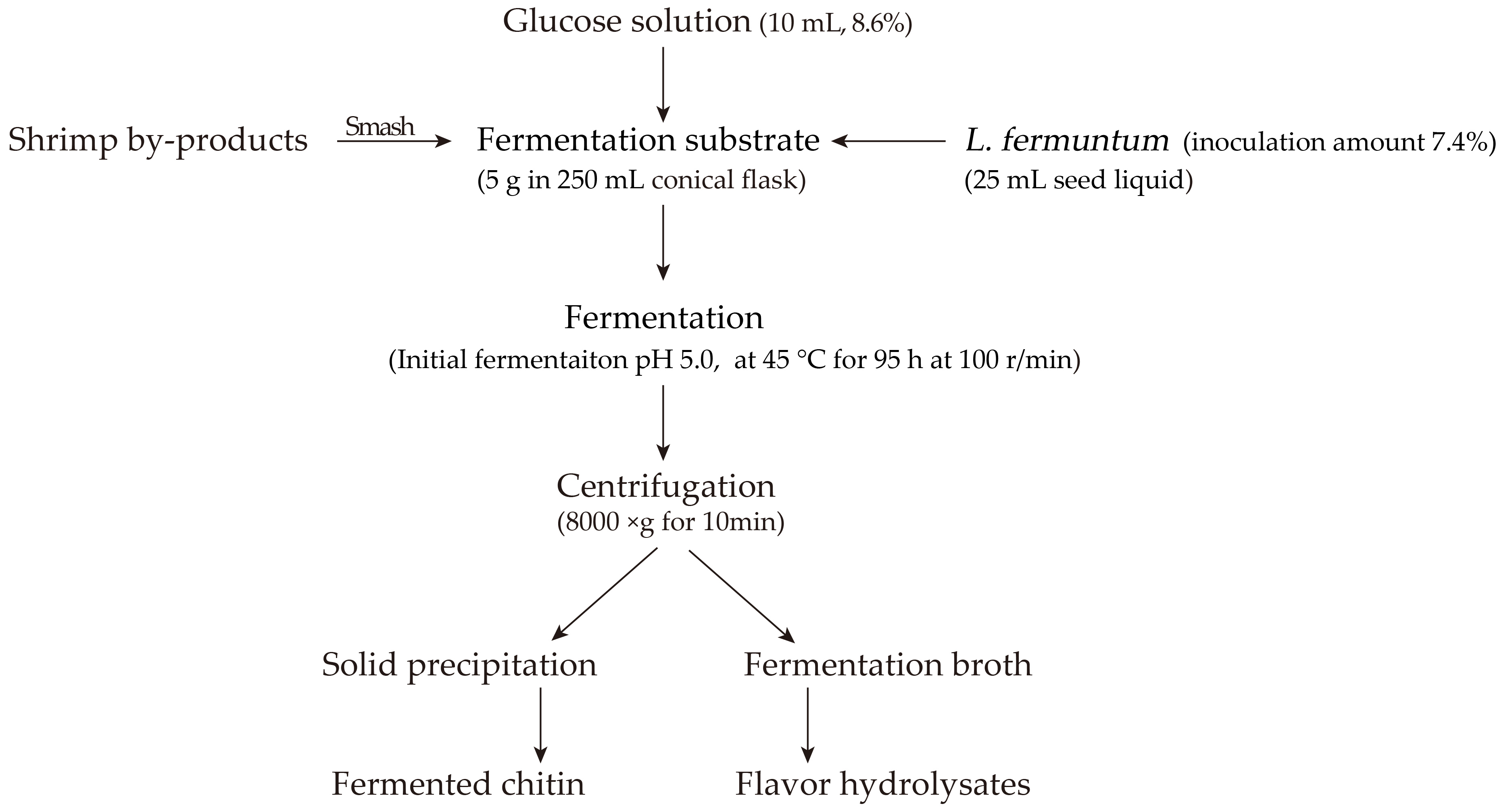

Supplement: Supplementary file 1 [file molecules-28-03761-s001.zip › Figure S2.jpg]
